# Commentary: As the Oceans Go, So Too Do We

**DOI:** 10.5334/aogh.3148

**Published:** 2020-12-03

**Authors:** Keith Martin

**Affiliations:** 1 Consortium of Universities for Global Health, US

We are using the planet’s oceans as a dumping ground. Waste from cities, agriculture, mining, other industries and marine vessels are pouring into marine ecosystems causing untold damage. Despite covering 70% of the planet’s surface and being a critical source of food, climate mitigation, biodiversity, employment and cultural goods, ocean pollution is an ignored global health threat that worsens by the day. As the oceans go, so too do we, for our health and that of the Earth’s oceans are inseparable.

This seminal report, ‘Human Health and Ocean Pollution’, led by Dr. Philip Landrigan of Boston College and Drs. Patrick Rampal and Hervé Raps of the Centre Scientifique de Monaco is timely and vital. It has been released in the midst of repeated warnings by leading scientific organizations and UN agencies on the destruction of Earth’s ecosystems as a consequence of human activity. The International Panel on Climate Change, UN Biodiversity, UN Environment, the Intergovernmental Platform on Biodiversity and Ecosystem Services, the Secretary-General of the United Nations and others have been vigorously warning the international community that we need to take urgent action to stop the destruction we are inflicting on our planet’s ecosystems [1,2,3,4,5]. We are causing this crisis and it is up to us to fix it.

This report clearly lays out the sources of pollution and the multiple impacts they are having on the oceans and on our health. It also provides a roadmap for policymakers, nongovernmental organizations and the public on the actions we need to take to stop this assault on our natural world.

To understand the daunting challenges we have created, we must first understand how profoundly our lives rest on the health of the world’s oceans. The report describes the ecosystem services oceans provide: food, livelihoods, cultural benefits, essential medications, mitigation of climate change and as a foundation of life on Earth. It appropriately outlines the powerful impact increasing greenhouse gas emissions are having on sea temperatures, ocean acidification, currents, weather patterns, marine biodiversity, fish stocks, and coral health. It lays out the devastating long-term effects of heavy metals, PCBs, and an array of other pollutants, the impact of which we only partially understand. The gap in our knowledge of the effects these substances are having on our health and environment is enormous. Tens of thousands of chemicals are already in our environment and the more than 1000 new chemicals are approved for use every year. The overwhelming majority of these are not fully tested for their safety.

This report is not a list of problems. It lays out recommendations that policymakers, nongovernmental organizations, communities, and individuals can implement to reverse the damage we are doing. It links rigorous science to policies that have been proven to work.

Enric Sala, a former university professor, marine ecologist and *National Geographic* Explorer-in-Residence was once asked why he created the Pristine Seas Program in 2008 [6]. He said he did not want to write the obituary of ocean life and decided to become a full-time conservationist. Since the creation of Pristine Seas, he and his team have worked with countries around the world to create 22 Marine Protected Areas that protect more than 5,000,000 km². Follow up monitoring has shown that in many of these areas, ocean ecosystems are returning to health. This report in the *Annals of Global Health* calls for the designation of additional Marine Protected Areas which will safeguard critical marine ecosystems, protect vulnerable fish stocks, and enhance human health and well-being.

Nature can heal itself, but we are running out of time. This report is a clarion call to save the planet’s oceans and by extension, save ourselves. Its findings need to be implemented. Our lives depend on it.
